# Plasma Proteomic Signature in Overweight Girls Closely Correlates with Homeostasis Model Assessment (HOMA), an Objective Measure of Insulin Resistance

**DOI:** 10.4061/2011/323629

**Published:** 2011-10-06

**Authors:** Stephen W. Rothwell, Merrily Poth, Harkirtin McIver, Chiedozie Ayika, Ofer Eidelman, Catherine Jozwik, Harvey B. Pollard

**Affiliations:** 1 Department of Anatomy, Physiology and Genetics, Uniformed Services University of the Health Sciences, Bethesda, MD 20814-4799, USA; 2 Departments of Pediatrics, Uniformed Services University of the Health Sciences, Bethesda, MD 20814-4799, USA

## Abstract

Obesity is known to be associated with a large number of long-term morbidities, and while in some cases the relationship of obesity and the consequences is clear (for example, excess weight and lower extremity orthopedic problems) in others the mechanism is not as clear. One common system of categorizing overweight in terms of the likelihood of negative consequences involves using the concept of “metabolic syndrome”. We hypothesized that the development of a plasma protein profile of overweight adolescents with and without the metabolic syndrome might give a more precise and informative picture of the disease process than the current clinical categorization and permit early targeted intervention. For this paper, we used antibody microarrays to analyze the plasma proteome of a group of 15 overweight female adolescent patients. Upon analysis of the proteome, the overweight patients diverged from the nonoverweight female controls. Furthermore, the overweight patients were divided by the analysis into two population clusters, each with distinctive protein expression patterns. Interestingly, the clusters were characterized by differences in insulin resistance, as measured by HOMA. Categorization according to the presence or absence of the metabolic syndrome did not yield such clusters.

## 1. Introduction

The term metabolic syndrome is used to describe a collection of factors associated with increased cardiovascular morbidities [1–10]. These risk factors can be clinically assessed by conventional physical examination and laboratory tests. The abnormalities can be grouped into the areas of obesity, lipid dysregulation, insulin resistance, and cardiovascular abnormalities [11–13]. The most straightforward measurement is the level of obesity which is usually quantified as the body-mass index (BMI), although waist measurement or waist/hip ratios are also used to define risk [14–16]. Dyslipidemia is defined as increased triglycerides, decreased high-density lipoprotein (HDL) cholesterol concentrations in the blood [17–19], and hypertension [20–22]. Decreased sensitivity to insulin is perhaps the single central characteristic of the syndrome [23–26]. Resistance to insulin effects on glucose metabolism may range from mild to severe. Other factors seen in patients with the syndrome that contribute to cardiovascular pathology include the proinflammatory and procoagulatory states displayed by many affected individuals. While common, these factors are not usually included in the strict definition of metabolic syndrome.

The incidence of obesity continues to rise, reaching 50% or greater in many populations. Based on the current definition of metabolic syndrome, it was estimated in 2002 by Ford et al. (Third National Health and Nutrition Examination Survey (NHANES), [11]) that greater than 25% of the American population could be considered to have metabolic syndrome. A 2009 evaluation of the NHANES 2003–2006 data [27] confirmed an ongoing increase in the numbers of affected individuals, with an overall percentage of Americans adults classified as having metabolic syndrome at the time of that survey, of 34%. This percentage increased with age, going from 20.3% for adults 20–39 years old to 40.8% for adults 40–59 years old and to 51.5% for adult over the age of 60 [27]. This is dismaying as it translates into twofold greater risk of death from cardiovascular complications as well as three times the likelihood of myocardial infarction and stroke for these individuals when compared to adults not diagnosed with metabolic syndrome. Perhaps even more dismaying, that same database estimated the incidence of obesity in adolescents in the USA as 30%. Based on this data, it has been estimated that the current generation of adolescents may be the first to have a shorter life expectancy than their grandparents. It is clear that identifying and understanding pathophysiologic factors leading to this grim projected consequence is an important mandate.

We hypothesized that an analysis of the plasma proteome from overweight girls might yield clues to the pathologic process, before secondary and tertiary consequences of the disorder were encountered. We further hypothesized that the proteome from overweight subjects with the metabolic syndrome would differ from those without the complete syndrome.

To test these hypotheses, we examined the proteomic signature of plasma from overweight girls, some with and some without the clinical characteristics of the metabolic syndrome. We hoped that this would provide relevant information on the disease process and might lead to novel avenues of intervention and treatment.

## 2. Methods

### 2.1. Plasma Preparation

Blood from obese female adolescent patients and healthy adult volunteers was collected in accordance with a human use protocol approved by the Institutional Review Boards of the Walter Reed Army Medical Center, Washington, DC and the Uniformed Services University of the Health Sciences, Bethesda, MD. Informed consent was obtained from parent or guardian and assent from the subjects themselves. To avoid possible interference of lipid levels with subsequent assays, blood collection was performed on fasting patients. Blood was collected using a 21 gauge butterfly needle into vacutainer tubes containing Acid Citrate-Dextrose as the anticoagulant. Prostacyclin (Sigma-Aldrich, St. Louis, MO) was added to the whole blood to a final concentration of 50 ng/mL (stock solution was 50 ug/mL in 50 mM Tris, 100 mM NaCl, pH 12, stored at −80°C until use) to remove unactivated platelets. The blood was first centrifuged at 400 RCF for 15 min at 23°C to pellet the erythrocytes and leukocytes. The platelet-rich plasma (PRP) was then removed, and prostacyclin was again added to the PRP. The PRP was then centrifuged at 1800 RCF for 20 min at 23°C to pellet the platelets. The plasma was removed and frozen in liquid nitrogen until analyzed.

### 2.2. Clinical Assessment of Adolescent Volunteers

Overweight adolescents presenting for evaluation and treatment were recruited for this study as part of a larger study of adolescent obesity. The definition for metabolic syndrome was based on BMI, HOMA, triglyceride levels, blood pressure, and HDL levels. Subjects that met three or more of the following criteria were classified as having metabolic syndrome: BMI greater than 85th percentile for age (see CDC website http://www.cdc.gov/healthyweight/assessing/bmi/childrens_bmi/about_childrens_bmi.html and Freedman et al. [28]); HOMA greater than 3.16 or fasting glucose greater than 109 mg/dL or 2-hour oral glucose test greater than 139 mg/dL; triglycerides greater than 95th percentile for age; blood pressure greater than 95th percentile for age and height [29] or HDL less than 40 mg/dL.

### 2.3. Antibody Microarray Assay

Protein profiles of patient and control plasma samples were analyzed with Clontech Antibody Microarray 500 (Mountainview, CA). Each slide contains 507 antibodies printed in duplicate (see website: http://bioinfo.clontech.com/abinfo/initialize.do#; cat # 63q790, lot # 8123984, for complete list of antibodies printed). Plasma samples were labeled with CY3 (GE Healthcare, CA) and a bronchial epithelial cell line that was used as a quality control standard was labeled with CY5 (GE Healthcare). Excess dye was removed with PD10 desalting columns. The samples were combined in a ratio of 80 ug plasma: 20 ug cell lysate and incubated with the Clontech microarray according to manufacturer directions (Clontech protocol PT3648-1). More protein was used for the plasma samples than the lysate because of the high proportion of protein in the plasma that was albumin. Following labeling, the slides were scanned in a Perkin Elmer ScanArray HT microarray scanner (Waltham, MA) at 90% laser power, 50 V PMT, and focus position 0.

### 2.4. Bioinformatics for Analysis of Antibody Microarrays

Data from scanned microarrays were loaded into the Perkin Elmer Express analysis software. Markers and control spots were removed. Only spots with a signal-to-noise ratio greater than 2 were used. Any duplicate spots that were different by greater than 20% were discarded (approximately 16% of total spots). The remaining spots were normalized to the median, and the ratio of plasma/control lysate for each antibody was calculated. This ratio was used to compare the relative abundance of each protein between samples.

Two software tools were used to analyze the protein expression data. First, cluster analysis was performed to determine hierarchical clustering and groupings of samples with similar levels of protein expression (Cluster and Treeview, MB Eisen, http://rana.lbl.gov/EisenSoftware.htm). Unsupervised clustering analysis divided the donors into three groups: controls and two sets of patients. Using these groupings, pathway analyses and examination of interactions between the patient protein sets were performed using Ingenuity Pathway Analysis (Ingenuity Systems, Inc., Redwood City, CA). Patient/control ratios were calculated for each protein on the array and the two patient groups were compared for expression in the canonical pathways.

For this pilot study, only three control samples were analyzed. This is a low number of samples, and, for future work, the goal is to analyze 100 patient and 100 age-matched control plasmas.

## 3. Results

### 3.1. Patient Demographics

Fifteen overweight adolescent girls with ages ranging from 13 to 17 years old (14.6 ± 1.5 y.o.) were recruited for the study. Their BMI ranged from 26.7 to 45.5 (33.8 ± 5.4). Exclusion criteria included frank diabetes or the use of any drug known to affect blood pressure, lipid profile, or carbohydrate metabolism. All subjects were otherwise in good health. The demographic information is summarized in Table 1. From these data, we identified three clinical subgroups: (i) female controls (FC), (ii) obese adolescent females displaying fewer than three of the accepted metabolic syndrome criteria (Fob), and (iii) obese adolescent females with three or more of the metabolic syndrome criteria (Fms).

**Table 1:** Patient demographics. Study subjects were recruited from patients referred to the Pediatric endocrinology clinic at the Walter Reed Army Medical Center. Patients entered into the study were given a routine health assessment and a Body Mass Index (BMI) was calculated. A glucose tolerance test was administered and insulin sensitivity/resistance was determined and calculated as HOMA value. Controls were normal adult women volunteers. FMS: female metabolic syndrome patient (3 or 4 factors), FOb: female obese patient (fewer than 3 factors), FC: female control.

Classification	Patient number	No. of factors	Age (years)	BMI	HOMA	HDL	TG
FMS	17	4	17	34.9	4.68	64	137
FMS	18	3	13	35.3	10.8	35	88
FMS	23	3	15	31.7	2.64	19	137
FOb	32	2	13	28.8	2.8	47	183
FOb	10	2	17	42.6	2.9	38	31
FOb	19	1	13	26.7	2.68	67	96
FOb	22	2	16	35.6	3.41	46	93
FOb	24	2	13	27.8	5.74	40	98
FOb	25	2	15	33.9	7.03	50	112
FOb	29	2	16	45.5	8.46	44	73
FOb	31	2	15	29	3.2	58	70
FOb	34	2	14	37.6	6.49	46	87
FOb	35	2	13	29	3.19	56	61
FOb	36	2	13	36	7.23	53	69
FOb	38	1	16	32.9	3.01	68	92
FC	3	1	38	21.3	Na	Na	Na
FC	5	1	34	20.6	Na	Na	Na
FC	8	1	33	Na	Na	Na	Na

### 3.2. Supervised Proteomic Analysis, Based on Preidentified Clinical Subgroups

Table 2 identifies proteins that are distinct to each of the three identified clinical subgroups referred to in the demographic descriptions. The analytic workflow consists of the following steps: (i) Firstly, the ratio of the patient sample/control cell lysate for each of 507 antibodies on the array is calculated to give a unique value for that specific protein. (ii) Secondly, the values are then averaged for the patients within each of the three clinically defined sub-groups. (iii) Thirdly, the ratio of Fob/Fc and Fms/Fc for each antibody is calculated to assess the average difference in expression for each protein between the groups. (iv) Finally, using Ingenuity Pathways Analysis software (IPA), the data are analyzed by searching for proteins that exhibit increased expression in only the Fob/Fc comparison or only the Fms/Fc comparison. The rationale is that this protein profile would help define the differences between patients who are obese, but do not have the complete metabolic syndrome, and patients who fit the clinical definition of metabolic syndrome. Using this methodology, Table 2 shows that 21 unique proteins can be identified that distinguish Fob from Fc, and 29 proteins can be identified that can distinguish Fms from Fc. The supervised approach has the advantage of identifying those proteins whose expressions most correlate with clinical impression.

**Table 2:** Study subjects were classified as either obese but not metabolic syndrome or obese with metabolic syndrome based on the clinical findings and compared to the control subjects. Proteins were identified that had expression levels that were at least 1.5-fold different between the controls and the obese patients or between controls and metabolic syndrome patients (either upregulated or downregulated). Table 2(a) shows proteins with expression levels significantly altered only in FMS compared to FC. Table 2(b) shows proteins with expression levels significantly altered only in Fob compared to FC. Table 2(c) shows proteins with expression levels significantly altered only in both FMS and Fob compared to FC. Fold changes and *P* values relative to the controls are listed.

(a)			

Fold change		Entrez gene name	*P*-value
3.192		Interleukin 3 (colony-stimulating factor, multiple)	3.49*E* - 02
2.404		Interleukin 13	3.36*E* - 02
2.133		Caspase 9, apoptosis-related cysteine peptidase	1.21*E* - 02
2.13		Protein kinase N2	1.12*E* - 02
2.046		Adenomatous polyposis coli	6.91*E* - 03
1.976		Disabled homolog 2, mitogen-responsive phosphoprotein (Drosophila)	8.04*E* - 03
1.799		Nuclear factor of activated T cells, cytoplasmic, calcineurin-dependent 1	6.39*E* - 03
1.764		RAS p21 protein activator (GTPase activating protein) 1	4.90*E* - 03
1.732		Caldesmon 1	3.24*E* - 02
1.714		RAP1A, member of RAS oncogene family	3.13*E* - 03
1.647		Nonmetastatic cells 1, protein (NM23A) expressed in	1.66*E* - 02
1.604		Diablo homolog (Drosophila)	4.60*E* - 02
1.572		GRB2-related adaptor protein 2	1.90*E* - 02
−1.619		Signal transducer and activator of transcription 6, interleukin-4 induced	3.57*E* - 06
−1.621		Epidermal growth factor receptor (erythroblastic leukemia viral (v-erb-b)	1.48*E* - 03
−1.628		Reversion-inducing-cysteine-rich protein with kazal motifs	2.57*E* - 03
−1.635		Tumor necrosis factor (TNF superfamily, member 2)	2.81*E* - 03
−1.637		Tight junction protein 1 (zona occludens 1)	8.31*E* - 05
−1.655		V-erb-b2 erythroblastic leukemia viral oncogene homolog 2	1.37*E* - 02
−1.722		Mitogen-activated protein kinase kinase 5	1.57*E* - 02
−1.724		Conserved helix-loop-helix ubiquitous kinase	1.64*E* - 03
−1.736		Tumor protein p53	6.75*E* - 03
−1.842		Chromogranin A (parathyroid secretory protein 1)	2.27*E* - 04
−1.842		Eukaryotic translation initiation factor 4E	1.16*E* - 03
−1.913		Minichromosome maintenance complex component 6	8.38*E* - 05
−1.929		Glutamate receptor, ionotropic, N-methyl D-aspartate 2B	4.98*E* - 07
−2.052		Centrosomal protein 250 kDa	9.46*E* - 05
−2.142		Caspase 4, apoptosis-related cysteine peptidase	2.48*E* - 08
−3.224		Diaphanous homolog 1 (Drosophila)	2.80*E* - 03

(b)			

Fold change		Entrez gene name	*P*-value

2.249		Myeloid cell leukemia sequence 1 (BCL2-related)	4.93*E* - 02
1.896		Retinoblastoma-like 2 (p130)	3.05*E* - 07
1.769		Caveolin 1, caveolae protein, 22 kDa	4.10*E* - 06
1.728		DEAD (Asp-Glu-Ala-Asp) box polypeptide 1	7.47*E* - 05
1.725		Protein kinase C, beta	7.56*E* - 07
1.716		E2F transcription factor 1	5.22*E* - 04
1.69		CD28 molecule	7.36*E* - 04
1.651		Son of sevenless homolog 1 (Drosophila)	1.16*E* - 03
1.617		Colony stimulating factor 1 receptor	2.19*E* - 06
1.588		Protein tyrosine phosphatase, nonreceptor type 6	1.03*E* - 03
1.574		A kinase (PRKA) anchor protein 1	4.69*E* - 06
1.539		General transcription factor Iii	7.23*E* - 05
1.536		Casein kinase 1, epsilon	8.53*E* - 03
1.535		Junction plakoglobin	5.09*E* - 08
1.509		Adaptor-related protein complex 3, mu 1 subunit	2.96*E* - 03
1.502		Cyclin-dependent kinase inhibitor 1A (p21, Cip1)	4.90*E* - 04
1.501		Proteasome (prosome, macropain) 26S subunit, ATPase, 5	5.18*E* - 03
1.729		Platelet-derived growth factor receptor, beta polypeptide	1.30*E* - 02
−1.512		Cyclin-dependent kinase inhibitor 1C (p57, Kip2)	5.60*E* - 03
−1.553		Chromodomain helicase DNA-binding protein 3	3.57*E* - 03
−1.686		Glycogen synthase kinase 3 beta	3.13*E* - 03

(c)			

Fold change		Entrez gene name	*P*-value

Fob	FMS		

2.301	−1.578	Androgen receptor	3.84*E* - 03
2.133	1.814	Insulin-like growth factor binding protein 3	1.31*E* - 02
1.95	2.13	Protein kinase N2	9.92*E* - 03
1.746	2.398	Transcription elongation regulator 1	4.43*E* - 03
1.622	1.614	SWI/SNF related, matrix associated, actin-dependent regulator of chromatin	6.15*E* - 03
1.612	1.875	Erythrocyte membrane protein band 4.9 (dematin)	1.08*E* - 03
1.608	−1.722	Mitogen-activated protein kinase kinase 5	5.09*E* - 05
1.554	−1.684	Interleukin 1, beta	5.54*E* - 04
−1.862	−1.691	Ribosomal protein S6 kinase, 70 kDa, polypeptide 1	2.22*E* - 06
−13.41	−5.309	Spleen focus forming virus (SFFV) proviral integration oncogene spi1	3.92*E* - 06

### 3.3. Unsupervised Proteomic Analysis, without Reference to Preidentified Clinical Subgroups

**Figure 1:**
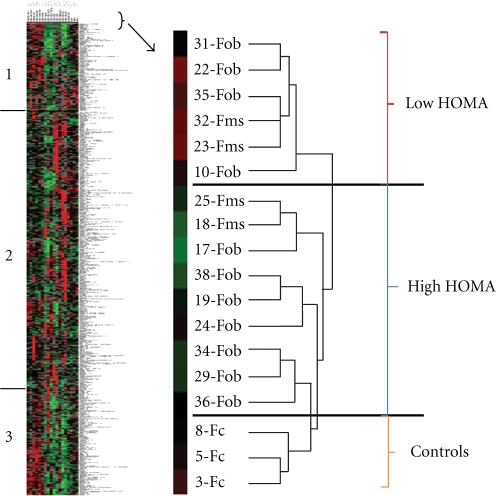
Cluster diagram representing plasma protein expression levels of female patient and control donors. Unsupervised clustering (Figure 1) separated the patients into two groups. Comparison of the patients in these groups to the clinical values revealed that the clustering correlates well with the HOMA values. Patients that clustered in what we designated the High HOMA group had HOMA values greater than 4.0 except for patients nos. 19 and 38 (7/9). All patients in the Low HOMA group had values less than 4.0. Based on a visual inspection on the cluster diagram, two regions of the protein set stood out as showing particular distinction between the groups. These proteins are in the two regions marked region 1 and region 2 on the diagram. Positive values represent binding increased relative to the protein standard and show up as red on the diagram, while values that are negative represent decreased protein binding and show up as green on the diagram.

An alternative approach is to determine if there is a specific proteomic signature that distinguishes between patients with metabolic syndrome, and those patients that are simply obese. Our strategy has therefore been to subject the data to analysis using a conventional hierarchical cluster algorithm [30], without consideration for any of the clinical information associated with each patient. Figure 1 shows data analyzed in this manner. Examination of the results shows that the control samples separate into a grouping that is distinct from the obese patient samples. The samples identified as metabolic syndrome patients do not form a separate grouping although the Fms samples do form two closely placed pairs. However, samples with high homeostatic model assessment (HOMA) values, regardless if they are identified as Fob or Fms, do separate from the patients with low HOMA. HOMA is the product of glucose and insulin concentrations, normalized to a constant, and is a conventional measure of insulin resistance [31]. The regions labeled 1 and 3 on the cluster diagram illustrate very clearly how the protein expression levels of the proteins separate the patient population into two distinct populations. The proteins contained in segment 1 are shown in Table 3. Together, the proteins in those two segments included 201 of the 507 proteins surveyed on the microarray. This alternative, unsupervised approach to identifying a proteomic signature for metabolic syndrome therefore suggests that insulin resistance may be a unique factor, correlating with a specific pattern of protein expression.

**Table 3:** Protein composition of region 1 from the cluster diagram (Figure 2). Unsupervised clustering (Figure 1) separated the patients into two groups. Based on a visual inspection on the cluster diagram, two regions of the protein set stood out as showing particular distinctions between the groups. These proteins are in the two regions marked region 1 and region 3 on the diagram in Figure 1. The values listed are derived from the fluorescent values representing protein binding on the antibody microarray. Positive values represent binding increased relative to the protein standard and show up as red on the diagram, while values that are negative represent decreased protein binding and show up as green on the diagram. The values for the entire 507 proteins on the microarrays are displayed in supplemental Table 1S available online at doi:10.4061/2011/323629.

Unique ID	Protein name	Low HOMA	Diagram color	High HOM	Diagram color	Control	Diagram color
331	hILP/XIAP	0.3121	Red	−0.1620	Green		Red
4927	Nup88	0.2371	Red	−0.4194	Green	0.0196	Red
9001	HAP-1	0.2739	Red	−0.3215	Green	0.0214	Red
4288	Ki-67	0.1811	Red	−0.3101	Green	0.1144	Red
7124	TNFa	0.2171	Red	−0.3622	Green	0.0082	Red
6732	SRPK1	0.2904	Red	−0.2250	Green	0.1255	Red
2064	erbB-2/HER-2	0.2099	Red	−0.2043	Green	0.1434	Red
60412	rSec8	0.2539	Red	−0.3078	Green	0.0849	Red
6550	NHE-3	0.1830	Red	−0.2553	Green	0.3126	Red
8828	Neuropilin-2	0.2417	Red	−0.3104	Green	0.0044	Red
7157	p53	0.1745	Red	−0.2634	Green	−0.0183	Green
821	Calnexin	0.1376	Red	−0.2462	Green		Red
4301	AF6	0.0948	Red	−0.3060	Green	−0.1052	Green
1263	Fnk	0.1443	Red	−0.3505	Green	−0.0640	Green
1977	eIF-4E	0.2086	Red	−0.2965	Green	0.0760	Red
3265	Ras (Ha)	0.1629	Red	−0.3541	Green	0.0564	Red
5577	PKA rIIb	0.2566	Red	−0.1664	Green	−0.0637	Green
482	Na+/K+ ATPase b2	0.0594	Red	−0.3157	Green		Red
837	Caspase-4/TX ICH-2	0.1421	Red	−0.3325	Green	0.0000	Green
5922	Gap1m	0.0999	Red	−0.3312	Green	−0.0447	Green
2280	FKBP 12	0.0520	Red	−0.3316	Green	−0.0258	Green
8434	RECK	0.0555	Red	−0.2946	Green	−0.0702	Green
1263	PRK	0.0568	Red	−0.3597	Green	0.0095	Red
1113	Chromogranin A	0.0825	Red	−0.2857	Green	0.1098	Red
136319	V-1/myotrophin	0.2074	Red	−0.3630	Green	0.0316	Red
6778	Stat 6	0.3217	Red	−0.3598	Green	0.0146	Red
4087	Smad2	0.2301	Red	−0.1654	Green	−0.0160	Green
394	p190	0.2663	Red	−0.0975	Green		Red
160	Adaptin a	0.0971	Red	−0.3414	Green	0.1676	Red
3916	Lamp-1	−0.0467	Green	−0.1003	Green	0.3199	Red
8717	TRADD	0.1246	Red	−0.1444	Green		Red
4436	MSH2	−0.0851	Green	−0.1053	Green	0.2459	Red
53917	Rab24	−0.0712	Green	0.0840	Red	0.1473	Red
5079	Pax-5	−0.0179	Green	0.1400	Red	−0.0893	Green
997	CDC34	0.0751	Red	−0.2010	Green	0.0720	Red
5335	Phospholipase C *γ*1	0.0712	Red	−0.2652	Green	0.0816	Red
4331	p36	0.2287	Red	−0.1030	Green	0.0086	Red
10241	NDP52	0.3068	Red	−0.1806	Green	0.1283	Red
4149	Max	0.2633	Red	−0.0925	Green	−0.0140	Green
857	Caveolin 1	0.3876	Red	−0.1245	Green	−0.0466	Green
309	Annexin VI	0.3409	Red	−0.1283	Green	−0.0005	Green
6921	SIII p15	0.3574	Red	−0.0806	Green	−0.1393	Green
1870	E2F-2	0.2694	Red	−0.1281	Green	0.0770	Red
4841	p54nrb	0.2229	Red	−0.0289	Green	−0.1176	Green
1981	eIF-4g	0.1675	Red	0.0356	Red	−0.0793	Green
57448	BRUCE	0.1585	Red	−0.1595	Green	−0.0811	Green
940	CD28	0.2257	Red	−0.1958	Green	−0.1689	Green
4046	LSP-1	0.1895	Red	−0.2442	Green	0.0870	Red
891	Cyclin B1	0.2257	Red	−0.2798	Green	0.0347	Red
2752	Glutamine synthetase	0.2952	Red	−0.1749	Green	−0.0764	Green

### 3.4. Functional Analysis of the Insulin Receptor Signaling Pathway in Metabolic Syndrome Patients

**Figure 2:**
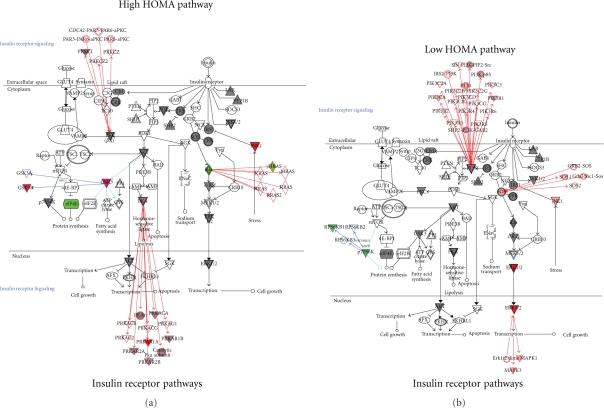
Canonical pathways for the insulin receptor. Protein expression levels for all patients were input into the Ingenuity Pathways Analysis software for analysis of changes in relevant pathways. IPA software has 272 annotated canonical pathways to which it will fit the experimental values of protein expression. Using this analysis function, it is possible to see if (1) the proteins of interest are involved in specific pathways and (2) how the expression levels of those proteins may affect the activity of the pathway. Proteins from our microarray data that are present in the canonical scheme are shaded. If the ratio of patient to control is less than 1.5, the shading of the protein symbol is gray. If the ratio is greater than 1.5, the symbol is red for an increase compared to the control and green for a decrease compared to the control. To limit the pathways to a manageable number, for this analysis, we examined the pathway of insulin action and the pathway of NF*κ*B activation and compared how the two groups of patients differed in their expression levels. The insulin receptor pathway was selected for display.

The unsupervised approach to data analysis suggests that insulin resistance plays a key role in structuring the proteomic signature for metabolic syndrome. We therefore used the expression levels of the proteins identified by the hierarchical cluster algorithm to investigate the insulin receptor signaling pathway in the metabolic syndrome patients. As shown in Figure 2, the pathway has three major branch points leading from the receptor. Initially ten signal transduction pathways lead out from the receptor. Two of these pathways, SOS (“son of sevenless”, Ras binding protein) and IRS1 (insulin receptor substrate), will lead to downstream transcription control. Furthermore, SHP2 (tyrosine phosphatase) and JNK1 (serine/threonine kinase) can inhibit the IRS1 transcription pathway. If IRS1 is not inhibited, this pathway will eventually activate AKT (serine/threonine kinase, also known as protein kinase B, PKB) and lead to transcription events that can influence cell growth or apoptosis. AKT also will inhibit and act on the mTOR and GSK3 pathways of protein synthesis. SOS will activate the Ras pathway which connects to MEK1/2 and ERK1/2 which will also mediate transcription and cell growth. The third major pathway leading from the insulin receptor connects to the glucose transporter GLUT4 pathway where the composition of lipid rafts and the mechanics of vesicle formation may be altered. Because all of the proteins from the antibody arrays are screened against the canonical pathways, both the low HOMA and the high HOMA groups had same elements in the insulin receptor pathway [23]. However, the high HOMA group had three proteins that were strongly depressed in expression compared to the controls (H-Ras, elF4E, and PKCH, see Table 3) and one that was strongly elevated (JNK1). In contrast, the low HOMA group showed slightly elevated SOS1, ERK1/2, and JNK1/2 and slightly depressed RPS6K.

### 3.5. Functional Analysis of the NF*κ*B Pathway in Metabolic Syndrome Patients

**Figure 3:**
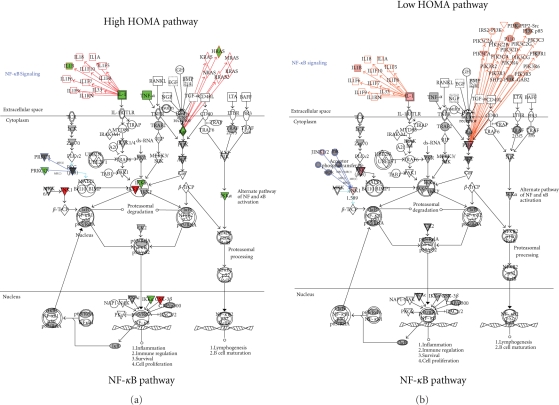
Canonical pathways for NF*κ*B. Changes in protein levels for High versus Low HOMA are shown as red for increased levels and green for decreased levels.

Examination of the NF*κ*B pathway (Figure 3) revealed strong differences between the high and low HOMA groups. Surprisingly, the molecules IL-1*β* and TNF-*α* were decreased relative to the controls in the high HOMA, whereas IL-1*β* was slightly elevated in the low HOMA set. From the stage of the IL-1*β* and TNF-*α* cell surface receptors, the signal is passed through a number of intermediate steps to phosphorylate the IKK complex. The phosphorylated IKK complex will, in turn, phosphorylate the inactive NF*κ*B complex. Our results show that the IKK subunits in the high HOMA were altered but were not changed in the low HOMA group. Changing the ratios of the various IKK subunits can be expected to modify the NF*κ*B complex processing, translocation to the nucleus and, ultimately, transcription patterns that could affect inflammation and immune regulation.

Selecting subsets of the larger pathway, such as the TNF-*α*/TNFR1 pathway or the IL-4 signaling pathway, allows further examination of details of NF*κ*B activation. For example, by reviewing the TNF-*α*/TNFR1 pathway, it was observed that IKK*α* decreased and IKK*β* increased in the high HOMA group but there are no significance changes in the low HOMA group. Another detail that is apparent with the higher resolution examination is that the apoptotic-inducing enzyme, caspase 9, is increased in the high HOMA group and not in the low HOMA group. Close examination of the IL-4 pathway also shows changes in NFATC 1 and 2 (nuclear factor for activated T cells) in the low HOMA group but not in the high HOMA group.

## 4. Discussion

These data show that a subset of overweight girls exhibits a specific proteomic signature in plasma, and that the discrimination made by the proteomic analysis is most closely associated clinically with the HOMA value. The potential importance of this finding is echoed by the results of analysis of the components of the insulin receptor pathways. If this characteristic, insulin resistance, is the most important factor dividing the overweight girls into two groups, it may be that we should be emphasizing the identification of insulin resistance as the primary goal in the evaluation of overweight adolescents. Further it may be that therapeutic interventions should be targeted specifically to this improving insulin sensitivity, rather than concentrating, as we do, on weight loss as the major goal of interventions. This is particularly important as there are interventions, such as exercise, that may improve insulin sensitivity without necessarily decreasing BMI.

### 4.1. Analysis of the Plasma Proteome Based on Clinical Criteria Alone

For the analysis in this paper, two approaches were employed. For the first approach, we grouped our patients according to clinical criteria. Study subjects were classified as either overweight without metabolic syndrome or overweight with metabolic syndrome based on the clinical findings. The two categories were then compared to the control subjects. Working on the hypothesis that differential protein expression could separate the two classes of obese subjects into distinct groups, the proteome was screened for proteins that were altered from the control values by at least 1.5-fold in the obese group or the obese/metabolic syndrome group, but not in both groups. This gave the two groups of proteins shown in Tables 2(a) and 2(b). Another analysis was conducted to see which altered proteins were common to both obese groups compared to the control samples. This is a shorter list with several proteins that suggest avenues for further research. For example, S6K (ribosomal protein S6 kinase) is substantially deceased in both obese groups. This protein is involved in the phosphorylation of IRS-1 (insulin receptor substrate-1) under the influence of TNF-alpha [32]. In rats and mice, it is proposed that this phosphorylation pathway leads to inflammation and insulin resistance in obesity [33, 34]. However, it may not be quite that straightforward since, in both patient groups, S6K was decreased. Another protein, SWI/SNF-related, matrix-associated, actin-dependent regulator of chromatin, is a coactivator/repressor along with Brd2, of pancreatic cell function [35]. Disruption of Brd2 function causes obesity in mice. Even seemingly unrelated proteins, such as spleen focus forming virus (SFFV) proviral integration oncogene spi1, may be connected to the problem of insulin resistance. In a study by Nishigaki et al., cells infected with spleen focus forming virus exhibited Akt kinase activation [36]. Akt is upstream in the signal transduction pathway of S6K.

A fourth protein that should be considered is the androgen receptor. Expression of the androgen receptor is altered in both groups. This seems like a reasonable event since it has been reported that testosterone levels in women (and men) can be greatly altered in the obese and metabolic status [37–40]. However, there seems to be a major gender difference in the influence of androgens. While testosterone is often elevated in obese women [39, 41], it is usually depressed in obese men [38]. We did not analyze data from obese men in this study. It is interesting that the mean levels of the androgen receptor are elevated in the obese group and decreased in the metabolic syndrome group. These examples illustrate how proteins that seem to be unrelated may be important in understanding the pathology of obesity.

### 4.2. Analysis of the Plasma Proteome without Clinical Bias

In the unbiased analysis, the data were analyzed without preconceived clinical groupings. The proteins were analyzed using an unsupervised clustering methodology. It was expected *a priori* that the Fob and FMS groups would cluster into two separate groups. However, this was not the case. The parameter that seemed to have the greatest correlation with the cluster results was the HOMA value. Seven out of nine patients in the group that we have designated, the High HOMA group, had values above 4.0. In contrast, all patients in the Low HOMA group had values less than 4.0. This is an arbitrary cut-off, but it serves to quantify a specific parameter that distinguishes the two groups that the clustering analysis recognizes.

One of the aspects of altered protein expression that was analyzed by this approach was the change in the insulin receptor pathways. Although metabolic syndrome is a diagnosis based on multiple factors, Grundy and colleagues [42, 43], among others, have proposed that insulin resistance alone can provide a mechanism for most of the consequences of metabolic syndrome. Analysis of three longitudinal studies by Cameron et al. [44] suggests that central obesity, as assessed by waist circumference, precedes the appearance of the other factors of metabolic syndrome. A more refined measure of obesity, the umbilical waist-to-height ratio, predicts the likelihood of insulin resistance and correlates well with elevated HOMA-IR (*r* = 0.58, *P* < 0.0001) in a study by Guntsche et al. [45]. Once insulin resistance is established, many of the criteria defining metabolic syndrome begin to manifest. For example, insulin resistance is associated with increased sympathetic nervous activity, but blunted sympathetic nervous system responsiveness [46]. This sympathetic nervous activity has been linked to left ventricular hypertrophy and may predict future renal injury [47]. Abnormalities in the cellular physiology and biochemistry of the endothelium of the vascular wall have also been attributed to insulin resistance. In studies of New Zealand obese mice, a strain with the characteristics of increased insulin resistance, increased visceral fat, and increased blood pressure, it was observed that nitric synthase activity was decreased. In addition, superoxide production was increased, and increased inflammatory Mac-3^+^ cell infiltration was measured [5]. For these reasons, the first of the pathways studied in detail was the insulin receptor signaling pathway. As outlined in Results (see Figure 3), binding of insulin to the insulin receptor initiates a number of phosphorylation events that include phosphorylation of the insulin receptor substrate, SHC adapter proteins, and the Grb2-associated binder-1 protein. One of the more important targets of insulin activation is phosphatidylinositol-3-kinase. Downstream events include Akt activation and the subsequent activation of nucleotide phosphodiesterase (PDE) which results in lower cAMP levels. Lowered cAMP levels lead to decreased activity of hormone sensitive lipase and decreased lipolysis. Thus, one can see that alterations can have effects at the level of modulation of energy stores and at the level of protein synthesis. The differences between the high HOMA group and the low HOMA are not large, but with a complex system such as the insulin receptor signal pathway, it can be imagined that small differences may be sufficient to disrupt normal regulatory processes to the extent that these changes have important clinical consequences.

## 5. Conclusions

Based on our analysis of this limited number of patients, it is clear that proteomic analysis of the plasma proteome of obese adolescents has the potential to yield valuable information concerning the underlying changes in the protein expression and metabolic changes that give rise to the phenotypic manifestations of the phenomenon known as metabolic syndrome. Our study is obviously limited by the small number of patients. The next challenge in this project is to increase the number of subjects and to widen the study to evaluate a larger cross-section of both female and male patients. Thus the data from this study, although promising, because of the small numbers should be considered preliminary and a project to extend and expand the investigation is warranted. The finding that the proteome signature appears to group subjects into those with and those without insulin resistance may be extremely important in understanding the pathophysiology of obesity in these young individuals. It is possible that interventions should be focused on increasing insulin sensitivity, for example, by increasing aerobic exercise and physical fitness, rather than the more difficult task of targeting significant weight loss.
